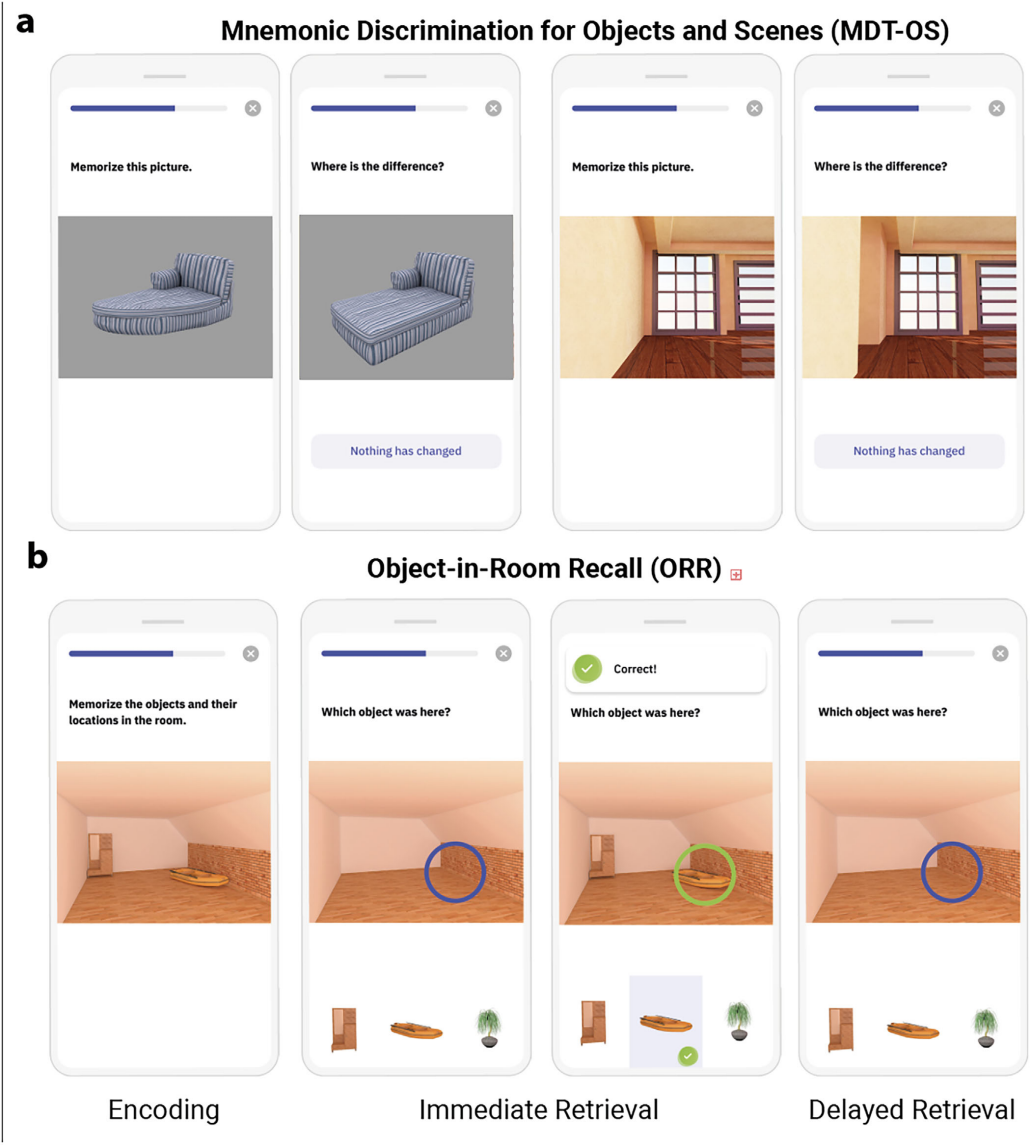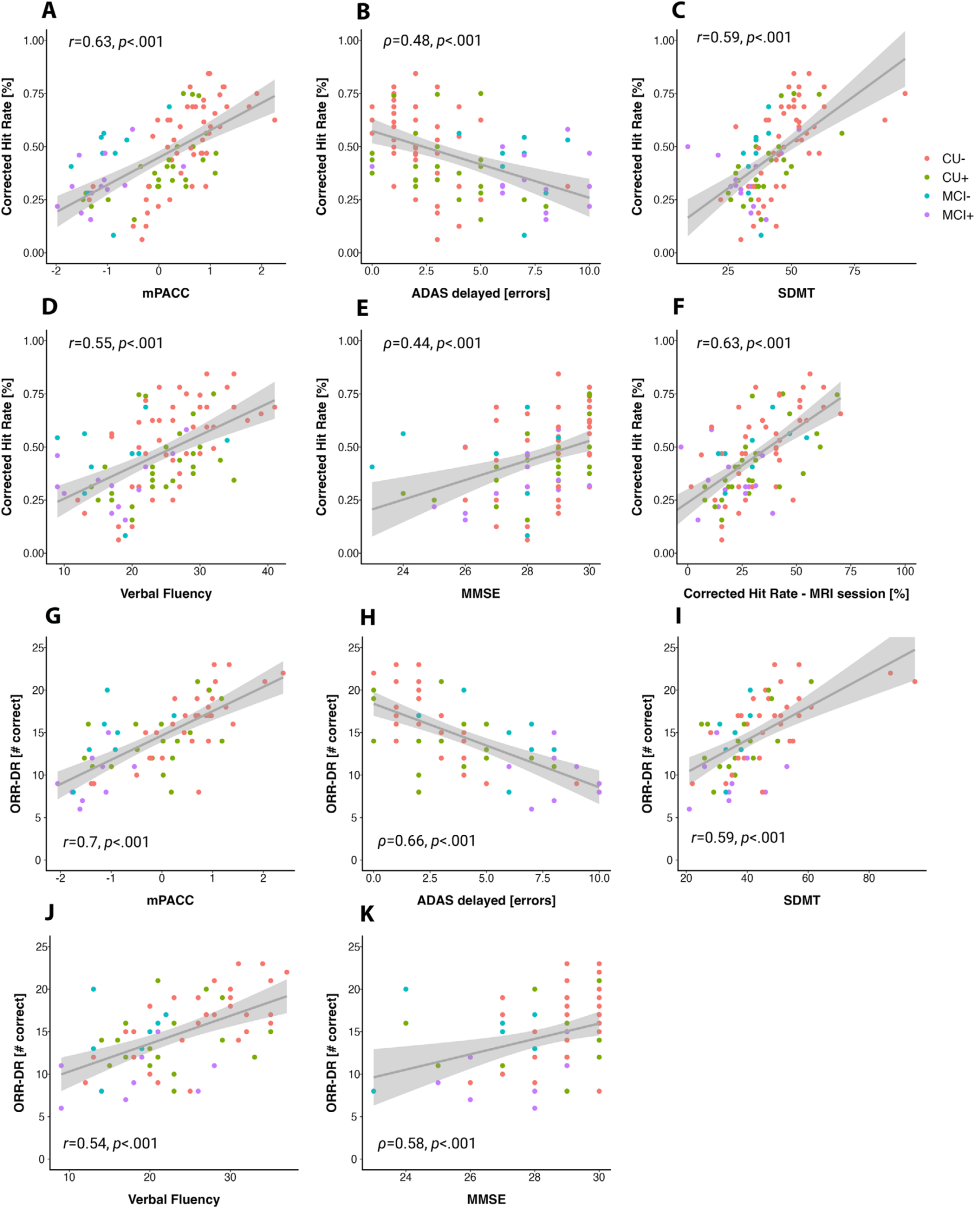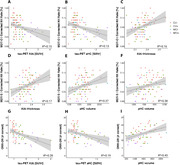# Remote and unsupervised digital memory assessments can reliably detect cognitive impairment in Alzheimer’s disease

**DOI:** 10.1002/alz.086091

**Published:** 2025-01-03

**Authors:** David Berron, Emil Olsson, Felix Andersson, Shorena Janelidze, Pontus Tideman, Emrah Düzel, Sebastian Palmqvist, Erik Stomrud, Oskar Hansson

**Affiliations:** ^1^ Clinical Memory Research Unit, Lund University, Lund Sweden; ^2^ German Center for Neurodegenerative Diseases (DZNE), Magdeburg Germany; ^3^ Clinical Memory Research unit, Lund University, Lund Sweden; ^4^ Memory Clinic, Skåne University Hospital, Malmö Sweden; ^5^ Clinical Memory Research Unit, Lund University, Malmö Sweden; ^6^ Institute of Cognitive Neuroscience, University College London (UCL), London United Kingdom; ^7^ Institute of Cognitive Neurology and Dementia Research (IKND), Otto‐von‐Guericke University, Magdeburg Germany; ^8^ Clinical Memory Research Unit, Department of Clinical Sciences Malmö, Faculty of Medicine, Lund University, Lund Sweden; ^9^ Clinical Memory Research Unit, Department of Clinical Sciences, Lund University, Lund Sweden; ^10^ Skåne University Hospital, Malmö, 21428 Skåne Sweden

## Abstract

**Background:**

Remote unsupervised cognitive assessments have the potential to complement and facilitate cognitive assessment in clinical and research settings.

**Method:**

Here we evaluate the usability, validity and reliability of unsupervised remote memory assessments via mobile devices (see Figure 1) in individuals without dementia from the Swedish BioFINDER‐2 study and explore their prognostic utility regarding future cognitive decline in combination with a plasma marker for p‐tau217.

**Result:**

Usability was rated positively. The app was easy to use with clear instructions, a majority of participants enjoyed completing the tests and preferred digital assessments over pen‐and‐paper tests, and more than 80% of participants found the frequency and length of remote assessments were appropriate. Remote memory assessments showed good construct validity with traditional neuropsychological assessments (see Figure 2) and were significantly associated with medial temporal lobe subregional tau‐PET and downstream MRI measures (see Figure 3). Memory performance at baseline was associated with future cognitive decline and prediction of future cognitive decline was further improved by combining remote digital memory assessments with plasma p‐tau217. Finally, retest reliability was moderate for a single assessment and good for an aggregate of two sessions.

**Conclusion:**

Our results demonstrate that unsupervised digital memory assessments might be used for diagnosis and prognosis in Alzheimer’s disease, potentially in combination with plasma biomarkers.